# Dysregulation of the (immuno)proteasome pathway in malformations of cortical development

**DOI:** 10.1186/s12974-016-0662-z

**Published:** 2016-08-26

**Authors:** J. van Scheppingen, D. W. M. Broekaart, T. Scholl, M. R. J. Zuidberg, J. J. Anink, W. G. Spliet, P. C. van Rijen, T. Czech, J. A. Hainfellner, M. Feucht, A. Mühlebner, E. A. van Vliet, E. Aronica

**Affiliations:** 1 Academic Medical Center, Department of (Neuro)Pathology, University of Amsterdam, Meibergdreef 9, 1105 AZ Amsterdam, The Netherlands; 2 Department of Pediatrics, Medical University Vienna, Vienna, Austria; 3 Department of Neurosurgery, Medical University Vienna, Vienna, Austria; 4 Department of Pathology, Medical University Vienna, Vienna, Austria; 5 Department of Pathology, Rudolf Magnus Institute for Neuroscience, University Medical Center Utrecht, Utrecht, The Netherlands; 6 Department of Neurosurgery, Rudolf Magnus Institute for Neuroscience, University Medical Center Utrecht, Utrecht, The Netherlands; 7 Swammerdam Institute for Life Sciences, Center for Neuroscience, University of Amsterdam, Amsterdam, The Netherlands; 8 Stichting Epilepsie Instellingen Nederland (SEIN), ᅟ, The Netherlands

**Keywords:** Immunoproteasome, Inflammation, Immunohistochemistry, Tuberous sclerosis complex, Focal cortical dysplasia, Epilepsy, Astrocytes, Inflammation

## Abstract

**Background:**

The proteasome is a multisubunit enzyme complex involved in protein degradation, which is essential for many cellular processes. During inflammation, the constitutive subunits are replaced by their inducible counterparts, resulting in the formation of the immunoproteasome.

**Methods:**

We investigated the expression pattern of constitutive (β1, β5) and immunoproteasome (β1i, β5i) subunits using immunohistochemistry in malformations of cortical development (MCD; focal cortical dysplasia (FCD) IIa and b, cortical tubers from patients with tuberous sclerosis complex (TSC), and mild MCD (mMCD)). Glial cells in culture were used to elucidate the mechanisms regulating immunoproteasome subunit expression.

**Results:**

Increased expression was observed in both FCD II and TSC; β1, β1i, β5, and β5i were detected (within cytosol and nucleus) in dysmorphic neurons, balloon/giant cells, and reactive astrocytes. Glial and neuronal nuclear expression positively correlated with seizure frequency. Positive correlation was also observed between the glial expression of constitutive and immunoproteasome subunits and IL-1β. Accordingly, the proteasome subunit expression was modulated by IL-1β in human astrocytes in vitro. Expression of both constitutive and immunoproteasome subunits in FCD II-derived astroglial cultures was negatively regulated by treatment with the immunomodulatory drug rapamycin (inhibitor of the mammalian target of rapamycin (mTOR) pathway, which is activated in both TSC and FCD II).

**Conclusions:**

These observations support the dysregulation of the proteasome system in both FCD and TSC and provide new insights on the mechanism of regulation the (immuno)proteasome in astrocytes and the molecular links between inflammation, mTOR activation, and epilepsy.

**Electronic supplementary material:**

The online version of this article (doi:10.1186/s12974-016-0662-z) contains supplementary material, which is available to authorized users.

## Background

The proteasome is an evolutionarily conserved multicatalytic proteinase complex representing a major protein degradation system, present in the nucleus and cytoplasm of eukaryotic cells, that regulates diverse biological processes essential for cell survival [1–4]. The proteolytic complex of the proteasome is represented by a catalytic 20S core particle, a barrel-shaped complex consisting of four heptameric rings, composed of non-identical α or β subunits [2, 5, 6]. The catalytic activity is restricted to three of the beta subunits β1, β2, and β5, which have specific cleavage preferences, and are constitutively expressed in most tissues. Under certain conditions associated with the release of immune-modulatory cytokines (i.e., interferon-γ, IFN-γ), a specialized type of proteasome called the immunoproteasome can be generated by the incorporation of the inducible subunits, β1i (PSMB9; LMP2, low molecular weight protein 2), β2i (PSMB10; LMP10, MECL-1, multicatalytic endopeptidase complex-like 1), and β5i (PSMB8; LMP7, low molecular weight protein 7) [7, 8]. Increasing evidence supports a key role of the immunoproteasome in the regulation of immune cell function, including both the adaptive and the innate immune response [9–11]. A deregulation of the immunoproteasome system, with induction of β1i and β5i subunits in the neurons and/or glial cells, has been reported in neurodegenerative diseases ([12–14] for review, see [15, 16]). In particular, recent studies have pointed to the role of immunoproteasome in glial cells, suggesting a novel interaction between immunoproteasome and glia-mediated inflammatory response, resulting in a pro-inflammatory environment [14, 16]. Interestingly, induction of β1i and β5i subunits has been also observed in specimens of patients with pharmaco-resistent mesial temporal lobe epilepsy (MTLE; [17]). Moreover, recent experimental data support a role for the β5i subunit in modulating seizure generation in epileptic tissue, and interestingly, this subunit was not upregulated in rats exposed to pilocarpine but not developing SE and spontaneous seizures [18].

In the present study, we investigated the expression and cellular distribution of both constitutive (β1, β5) and immunoproteasome (β1i, β5i) subunits using immunohistochemistry in a large cohort of patients with malformations of cortical development (MCD; including focal cortical dysplasia (FCD) type II and tuberous sclerosis complex (TSC) cortical tubers), evaluating a possible relationship between changes in the expression of these subunits and the clinical course of epilepsy. To provide better insights into the mechanisms underlying the astroglial regulation of immunoproteasome subunits, we studied their expression in response to IL-1β stimulation in both human fetal astrocytes and FCD-derived cells. Since both FCD II and TSC are associated with constitutive activation of the mammalian target of rapamycin (mTOR) pathway [19, 20], we further evaluated the effect of rapamycin (inhibitor of the mTOR pathway) in FCD II-derived cell cultures.

## Methods

### Subjects

The cases included in this study were obtained from the archives of the Departments of Neuropathology of the Academic Medical Center (AMC, University of Amsterdam, The Netherlands), the University Medical Center Utrecht (UMCU, The Netherlands), and the Medical University Vienna (MUV, Austria). A total of 23 brain tissue specimens, removed from patients undergoing surgery for intractable epilepsy, were examined. The tissue was obtained and used in accordance with the Declaration of Helsinki and the AMC Research Code provided by the Medical Ethics Committee and approved by the committee of the UMCU Biobank. This study was also approved by the Ethical Committee of the Medical University of Vienna. All cases were reviewed independently by two neuropathologists, and the diagnosis of FCD was confirmed according to the international consensus classification system recently proposed for grading FCD [21]. All patients with cortical tubers fulfilled the diagnostic criteria for TSC [22]. None of the FCD patients fulfilled the diagnostic criteria for TSC. Table 1 summarizes the clinical findings of patients with MCD and epilepsy (6 mild MCD (mMCD), 5 FCD IIa, 6 FCD IIb, 6 TSC tubers: 4 TSC2/2 TSC1; pre-operative seizure frequency/month, mean ± SEM: mMCD 19.8 ± 6.7; FCD II 149 ± 68.7; TSC 114.8 ± 24.2); seizure frequencies were recorded (video-electroencephalographic monitoring) at the time of the preoperative evaluation. One tuber specimen was obtained postmortem (age 32 years; male; TSC2). Hippocampal specimens from patients with Alzheimer’s disease (AD; *n* = 4; 3 females and 1 male; Braak stages V and VI, age 81.7 ± 2.8) were also examined as positive controls. In addition, normal-appearing control cortex and white matter were obtained at autopsy from six young adult control patients (Table 1), without history of seizures or other neurological diseases. All autopsies were performed within 24 h after death.

**Table 1:** Summary of clinical findings of epilepsy patients and controls

Pathology type	Number of cases	Gender (M/F)	Mean age (years/range)	Localization	Mean duration of epilepsy (years/range)
mMCD	6	5/1	21.5 (19–27)	3 fr/3 t	17.3 (11–32)
FCD IIa	5	3/2	34.2 (18–45)	4 fr/1 t	22.4 (14–26)
FCD IIb	6	2/4	33 (21–45)	4 fr/2 t	24 (15–40)
Cortical tubers (TSC)	6	3/3	7.1 (3–16)	4 fr/2 t	5.8 (0.8–13)
Controls/autopsy	14	8/6	27.0 (2–48)	6 fr/8 t	–

*FCD* focal cortical dysplasia, *TSC* tuberous sclerosis complex, *mMCD* mild malformations of cortical development, *M* male, *F* female, *fr* frontal, *t* temporal

### Tissue preparation and immunohistochemistry

Brain tissue from control and MCD patients was fixed in 10 % buffered formalin and embedded in paraffin. Paraffin-embedded tissue was sectioned at 5 μm, mounted on pre-coated glass slides (Star Frost, Waldemar Knittel GmbH, Braunschweig, Germany), and used for histology and immunohistochemistry. One representative paraffin block per case was sectioned, stained, and assessed. Sections were processed for hematoxylin eosin stainings, as well as for immunohistochemical stainings for a number of neuronal and glial markers and antibodies against the constitutive (β1, β5) and immunoproteasome (β1i, β5i) subunits (Table 2). These antibodies have been extensively tested on human liver and brain tissues [23, 24], including surgical brain specimens from patients with mesial temporal lobe epilepsy revealing bands at the expected molecular weight ([17]; Additional file 1: Figure S1). To detect differences in labeling related to technical variables such as tissue fixation, we also tested the antibodies in specimens of selected regions (temporal cortex/hippocampus) collected at autopsy and immediately fixed in formalin for 24 h (same fixation time used for the surgical specimens); no differences in the immunoreactivity pattern were observed.

**Table 2:** Immunohistochemistry: primary antibodies

Antigen	Primary antibody	Source	Dilution
Glial fibrillary acidic protein (GFAP)	Rabbit polyclonal	DAKO, Glostrup, Denmark	1:4000
Neuronal nuclear protein (NeuN)	Mouse clone MAB377	Chemicon, Temecula, CA, USA	1:2000
Phospho-S6 ribosomal protein (pS6)	Ser235/236; rabbit polyclonal	Cell Signaling Technology, Beverly, MA, USA	1:50
Interleukin 1β	Goat polyclonal	Santa Cruz Bio., Delaware CA, USA	1:70
MHC class I (HLA A, B, and C; HLA-I)	Mouse clone HC-10	^a^	1:200
MHC class II (HLA-DP, DQ, DR; HLA-II)	Mouse clone CR3/43	DAKO, Glostrup, Denmark	1:400
Proteasome β1	Mouse monoclonal IgG1	Enzo Life Sciences/Biomol, Farmingdale, NY, USA	1:200
Proteasome β5	Rabbit polyclonal	Enzo Life Sciences/Biomol	1:500
Proteasome β1i	Mouse monoclonal IgG1	Enzo Life Sciences/Biomol	1:200
Proteasome β5i	Mouse monoclonal IgG1	Enzo Life Sciences/Biomol	1:200

*MHC* major histocompatibility complex

^a^Gift from Prof. J. Neefjes, Netherlands Cancer Institute, The Netherlands

Single-label immunohistochemistry was performed as previously described [25]. Sections were deparaffinated in xylene, rinsed in ethanol (100, 95, and 70 %) and incubated for 20 min in 0.3 % hydrogen peroxide diluted in methanol. Antigen retrieval was performed using a pressure cooker in 0.1 M citrate buffer pH 6.0 at 120 °C for 10 min. Slides were washed with phosphate-buffered saline (PBS; 0.1 M, pH 7.4) and incubated overnight with the primary antibody in PBS at 4 °C. After washing in PBS, sections were stained with a polymer-based peroxidase immunohistochemistry detection kit (PowerVision Peroxidase System, ImmunoVision, Brisbane, CA, USA). The 3,3′-diaminobenzidine tetrahydrochloride was used as chromogen. Sections were dehydrated in alcohol and xylene and coverslipped.

Double-labeling of β1, β1i, β5, or β5i with NeuN (neuronal nuclear protein (NeuN; mouse clone MAB377; Chemicon, Temecula, CA, USA; 1:2000), GFAP (polyclonal rabbit, DAKO, Glostrup, Denmark; 1:4000, or monoclonal mouse, Sigma-Aldrich, St. Louis, MO, USA; 1:4000), HLA-I (mouse clone HC-10, 1:200), or HLA-II (mouse anti-human leukocyte antigen (HLA)-DP, DQ, DR, mouse clone CR3/43; DAKO; 1:400) was performed as previously described [26]). Sections were incubated with BrightVision poly-alkaline phosphatase (AP)-anti-rabbit or anti-mouse (Immunologic, Duiven, The Netherlands) for 30 min at room temperature and washed with PBS. AP activity was visualized with the AP substrate kit III Vector Blue (SK-5300, Vector Laboratories Inc., CA, USA). To remove the first primary antibody, sections were incubated at 121 °C in citrate buffer (10 mM NaCi, pH 6.0) for 10 min. Incubation with the second primary antibody was performed overnight at 4 °C. Sections with primary antibody other than rabbit were incubated with post-antibody blocking from the BrightVision+ system (containing rabbit-α-mouse IgG; Immunologic, Duiven, The Netherlands). AP activity was visualized with the alkaline phosphatase substrate kit I Vector Red (SK-5100; Vector Laboratories Inc., CA, USA). Sections incubated without the primary antibody, with preimmune sera, or with the antibody preincubated with the antigenic peptide (for the polyclonal β5) were essentially blank.

### Evaluation of histology and immunohistochemistry

All labeled tissue sections were evaluated by two independent observers for the presence or absence of various histopathological parameters and specific immunoreactivity (IR) for the different markers used for the diagnosis of mMCD, FCD subtypes, and TSC tubers. We also semi-quantitatively evaluated the IR (nucleus and cytoplasm in glial and neuronal cells) of β1, β1i, β5, and β5i. The intensity of the staining was evaluated using a scale of 0–3 (0: no; 1: weak; 2: moderate; 3: strong staining). All areas of the lesion were examined, and the score represents the predominant cell staining intensity found in each case. The frequency of β1, β1i, β5, or β5i positive cells ((1) rare; (2) sparse; (3) high) was also evaluated to give information about the relative number of positive cells within the lesion. We also evaluated intensity and frequency of pS6 and IL-1β staining. As described in previous studies [25, 27], the product of the intensity and frequency scores was taken to give the overall score (total score; immunoreactivity score (IRS), Table 3). Quantification of signal intensity using ImageJ software was performed for β1i and β5i subunits (Additional file 2: Figure S2).

**Table 3:** Immunoreactivity of β1, β1i, β5, and β5i proteasome subunits in the cortex

		(Dysmorphic) neurons		Glia		Balloon/giant cells	
		Cytoplasm	Nucleus	Cytoplasm	Nucleus	Cytoplasm	Nucleus
β1	Control	2 (2–3)	0	0 (0–1)	0	–	–
	mMCD	2▪°~	1 (1–2)*°~	0▪°~	0°~	–	–
	FCDIIa	5 (4–9)*	1 (1–2)*°	2.5 (2–4)*	2.5 (2–4)*°	–	–
	FCDIIb	7.5 (3–9)*	6 (3–6)*	4 (3–6)*	5 (4–6)*	3.5 (3–6)	6 (4–9)
	TSC	6.5 (4–9)*	5 (4–9)*	7.5 (4–9)*	7.5 (4–9)*	6 (3–9)	9 (6–9)
β1i	Control	0 (0–1)	0 (0–1)	1 (0–1)	0 (0–2)	–	–
	mMCD	0▪°~	0▪°~	0 (0–1)▪°~	0▪°~	–	–
	FCDIIa	3 (2–4)*	4 (3–6)*~	3.5 (3–4)*	3 (3–6)*~	–	–
	FCDIIb	4 (2–4)*	6 (3–9)*	6 (3–9)*	5 (3–6)*	4 (3–4)	7.5 (3–9)
	TSC	3.5 (2–6)*	6 (4–9)*	5 (3–9)*	5 (2–9)*	4 (3–6)	7.5 (4–9)
β5	Control	1 (0–1)	1 (0–1)	0	0 (0–1)	–	–
	mMCD	0°~	6*▪	0°~	0▪°~	–	–
	FCDIIa	3.5 (2–4)*~	7.5 (6–9)*	1.5 (0–2)*~	2.5 (1–4)*~	–	–
	FCDIIb	6 (4–6)*°	9 (6–9)*	3.5 (3–4)*	6 (4–9)*°	4 (3–6)	9 (6–9)
	TSC	3.5 (3–4)*	7.5 (6–9)*	2.5 (2–4)*	2.5 (2–4)*	6 (4–9)	9 (6–9)
β5i	Control	0	0	0 (0–1)	1 (1–2)	–	–
	mMCD	0°	0▪~	0 (0–1)°~	0°~	–	–
	FCDIIa	0°	1.5 (1–4)*	0 (0–2)°	0 (0–2)~	–	–
	FCDIIb	0 (0–1)°	4 (3–6)°	2.5 (1–3)°	3 (1–6)	0 (0–1)°	6 (4–6)°
	TSC	6 (4–9)*	0.5 (0–1)	6 (4–9)*	1.5 (1–2)*	9 (6–9)	0.5 (0–1)

Immunoreactivity score (IRS) is given as median (minimum-maximum). IRS is defined as intensity score multiplied by frequency score (see “Methods” section). Kruskall-Wallis test followed by Mann-Whitney *U* test

*Different compared to controls; °different compared to TSC; ▪different compared to FCDIIa; ~different compared to FCDIIb, *p* < 0.05

### Cell cultures

Primary fetal astrocyte-enriched cell cultures were obtained from human fetal brain tissue (14–19 weeks of gestation) obtained from the HIS-Mouse (human immune system mouse) facility of the AMC, Amsterdam. All materials have been collected from donors from whom a written informed consent for the use of the material for research purposes had been obtained by the Bloemenhove Clinic (Heemstede, The Netherlands); these informed consents are kept together with the medical record of the donor by the clinic. The tissue was obtained in accordance with the Declaration of Helsinki and the AMC Research Code provided by the Medical Ethics Committee of the AMC. Cell isolation was performed as described elsewhere [28–30]. Briefly, after the removal of the blood vessels, the tissue was mechanically minced into smaller fragments and enzymatically digested by incubating at 37 °C for 30 min with 2.5 % trypsin (Sigma-Aldrich; St. Louis, MO, USA). The tissue was washed with incubation medium containing Dulbecco’s modified Eagle’s medium (DMEM)/HAM F10 (1:1) medium (Gibco, Life Technologies, Grand Island, New York, USA), supplemented with 50 units/ml penicillin, 50 μg/ml streptomycin, and 10 % fetal calf serum (FCS; Gibco, Life Technologies, Grand Island, New York, USA) and triturated by passing through a 70 μm mesh filter. Cell suspension was incubated at 37 °C, 5 % CO_2_ for 48 h to let glial cells adhere to the culture flask before it was washed with PBS to remove excess of myelin and cell debris. Cultures were subsequently refreshed twice a week. Cultures reached confluence after 2–3 weeks.

Primary FCD astrocyte cultures were derived from a surgical human brain specimen obtained from a patient with FCD type IIA (age at surgery, 16 years; female; location, frontal; seizure frequency, thrice per week; duration of epilepsy, 11 years) undergoing epilepsy surgery at the Department of Pediatrics/Neurosurgery of the Medical University Vienna (Vienna, Austria). FCD astrocyte cultures were established in the same manner as described above for fetal cultures.

Secondary astrocyte cultures for experimental manipulation were established by trypsinizing confluent cultures and sub-plating onto poly-l-lysine (PLL; 15 μg/ml, Sigma-Aldrich)-precoated 12- and 24-well plates (Costar, Cambridge, MA, USA; 5 × 10^4^ cells/well in a 12-well plate for RNA isolation and PCR; 2.5 × 10^4^ cells/well for immunocytochemistry). In the present study, astrocytes were used for analyses at passages 2–4.

Cell cultures were stimulated with human recombinant (r)IL-1 β (PeproTech, Rocky Hill, NJ, USA; 10 ng/ml) or in some experiments with lipopolysaccharide (LPS; 100 ng/ml; Sigma-Aldrich, St. Louis, USA) for 24 h. Treatment of FCD-derived astrocytes with rapamycin (100 nM) was started 24 h before and continued during IL-1β stimulation. Cells were harvested 24 h after stimulation. Viability of human cell cultures was not influenced by the performed treatments (Additional file 3: Figure S3).

For immunofluorescent staining of cell cultures, sections were incubated with the primary antibodies for β1, β1i, β5, or β5i for 1 h at RT, followed by 2 h of incubation at RT with Alexa Fluor® 568-conjugated anti-rabbit or Alexa Fluor® 488-conjugated anti-mouse IgG (1:200, Molecular Probes, The Netherlands) together with Alexa Fluor® 488 or 594 Phalloidin (1:200, Molecular Probes, Plaats, The Netherlands) for counterstaining actin filaments. Sections were mounted using VECTASHIELD with DAPI (Vector Laboratories Inc., Burlingame, CA, USA). Fluorescent microscopy was performed using a Leica Confocal Microscope TSC SP-8X (Leica, Son, the Netherlands) at ×40 magnification (bidirectional X, speed 600 Hz, pinhole 1.00 AU).

### RNA isolation and real-time quantitative PCR analysis

For RNA isolation, cell culture material was homogenized in Qiazol Lysis Reagent (Qiagen Benelux, Venlo, The Netherlands). Total RNA was isolated using the miRNeasy Mini kit (Qiagen Benelux, Venlo, The Netherlands) according to the manufacturer’s instructions. The concentration and purity of RNA were determined at 260/280 nm using a NanoDrop 2000 spectrophotometer (Thermo Scientific, Wilmington, DE, USA). To evaluate β1, β1i, β5 or β5i, and IFNγ mRNA expression, 200 ng of cell-culture-derived total RNA was reverse-transcribed into cDNA using oligo dT primers. PCRs were run on a Roche LightCycler 480 thermocycler (Roche Applied Science, Basel, Switzerland) using the following primers: β1 (forward: accagctcggtttccaca, reverse: cccggtatcggtaacacatc); β5 (forward: gagtctcagtgatggtctgagc, reverse: actccatggcggaacttg); β1i (forward: accaaccggggacttacc, reverse: tcaaacactcggttcaccac); β5i (forward: ccctacccacccctgttt; reverse: cacccagggactggaaga); and IFN-γ (forward: gcaagatcccatgggttgtgt; reverse: ctggctcagattgcaggcata). Quantification of data was performed using the computer program LinRegPCR in which linear regression on the log (fluorescence) per cycle number data is applied to determine the amplification efficiency per sample [31, 32]. The starting concentration of each specific product was divided by the geometric mean of the starting concentration of the reference genes (EF1α and C1orf43), and this ratio was compared between groups.

### Statistical analysis

Statistical analyses were performed with GraphPad Prism software (Graphpad Software Inc., La Jolla, CA, USA). To assess differences in immunoreactivity score between multiple groups, non-parametric Kruskal-Wallis followed with Mann-Whitney *U* test was used. Correlations were assessed using Spearman’s (rho) rank correlation test. For cell culture data, Mann-Whitney *U* test was used to asses differences between different conditions. *P*<0.05 was assumed to indicate a significant difference.

## Results

### Case material and histological features

**Fig. 1:**
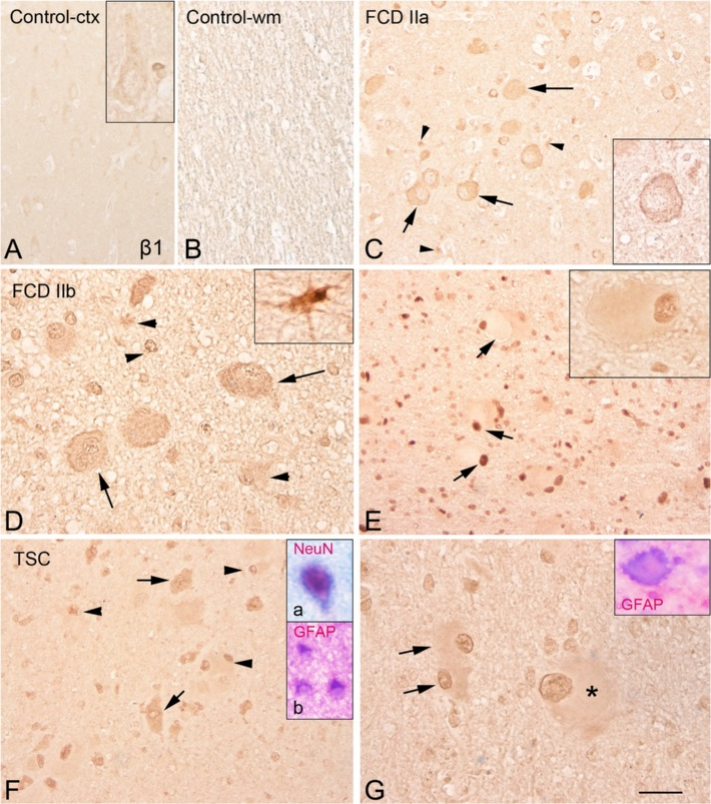
β1 proteasome subunit immunoreactivity in control, focal cortical dysplasia (FCD) type IIa, FCD type IIb, and tuberous sclerosis complex (TSC). Panels **a**, **b** (control) show weak immunoreactivity (IR) in both the cortex (**a** insert: neuron with weak expression of β1 subunit) and white matter (**b** not-detectable glial expression). Panel **c** (FCD IIa) shows positive dysmorphic neurons (*arrows*; insert: high magnification) and glial cells (*arrow heads*). Panels **d**–**e** (FCD IIb) show several β1 positive cells within the cortex (**d**) and white matter (**e**), including the dysmorphic neurons (*arrows* in **d**), glial cells (*arrow heads* and insert in **d**) and balloon cells (*arrows* in **e**; prominent nuclear expression; insert: high magnification). Panels **f**–**g** (TSC tuber): β1 subunit expression is observed within the tuber in dysmorphic neurons (*arrows* in **f**; insert a: co-localization with the neuronal marker NeuN; insert **b**, co-localization with GFAP), glial cells (*arrow heads* in **f**) and in giant cells (*asterisk* (**g**); insert: co-localization with GFAP). Additional examples of the different cell types at higher magnification in separate specimens. The inserts within panels show imagines of the different cells types at higher magnification in separate specimens. Scale bar in G: A–C, E–F: 80 μm; D–G: 40 μm

**Fig. 2:**
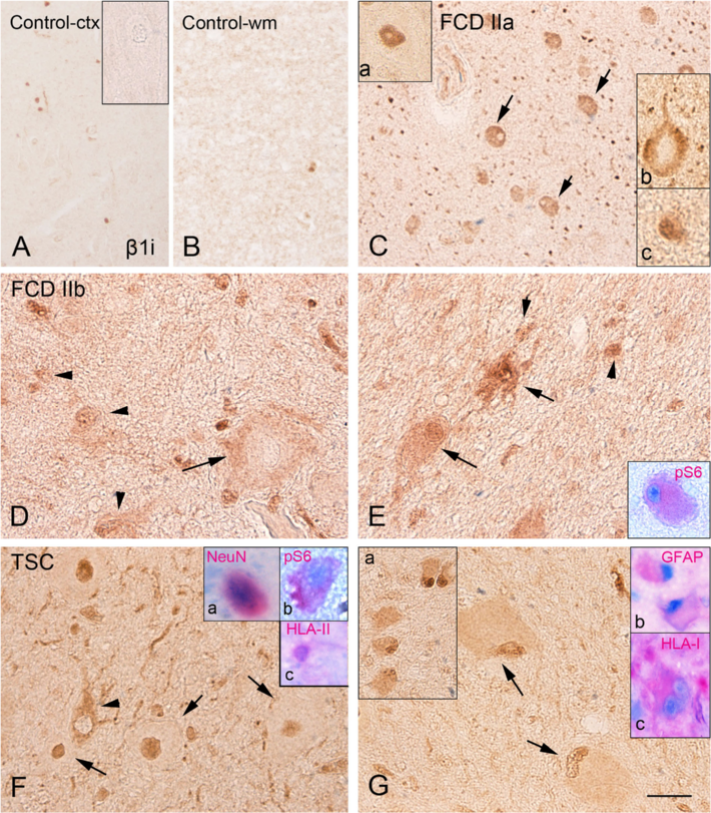
β1i proteasome subunit immunoreactivity in control, focal cortical dysplasia (FCD) type IIa, FCD type IIb, and tuberous sclerosis complex (TSC). **a**, **b** Control cortex **(a)** and with matter **(b)** with weak β1i expression (insert in **a**: negative neuron, high magnification). Panel **c** (FCD IIa) shows strong expression within the dysplastic region with positive dysmorphic neurons (*arrows*; inserts: **a** nuclear expression; **b** cytoplasmic expression) and glial cells (insert in **c**). Panels **d**–**e** (FCD IIb) show several β1i-positive cells within the cortex **(d)** and white matter **(e)**, including dysmorphic neurons (*arrow* in **d**), glial cells (*arrow heads* **d** and **e**), and balloon cells (*arrows* in **e**; nuclear and cytoplasmic expression; insert: co-localization with the pS6). Panels **f**, **g** (TSC tuber): β1i subunit expression is observed within the tuber in dysmorphic neurons (**f** *arrows*, nuclear expression; *arrow head*, cytoplasmic expression; insert **a** in **f**: co-localization with the neuronal marker NeuN; insert **b** in **f**: co-localization with the pS6; insert **c**: co-localization with HLA-II), glial cells (insert **b** in **g**), and in giant cells (*arrows* in **g**; insert **b**: co-localization with GFAP; insert **c**: co-localization with HLA-I). The inserts within the panels show images of the different cell types at higher magnification in separate specimens. Scale bar in **g**: **a–c**: 80 μm; **d–g**: 40 μm

**Fig. 3:**
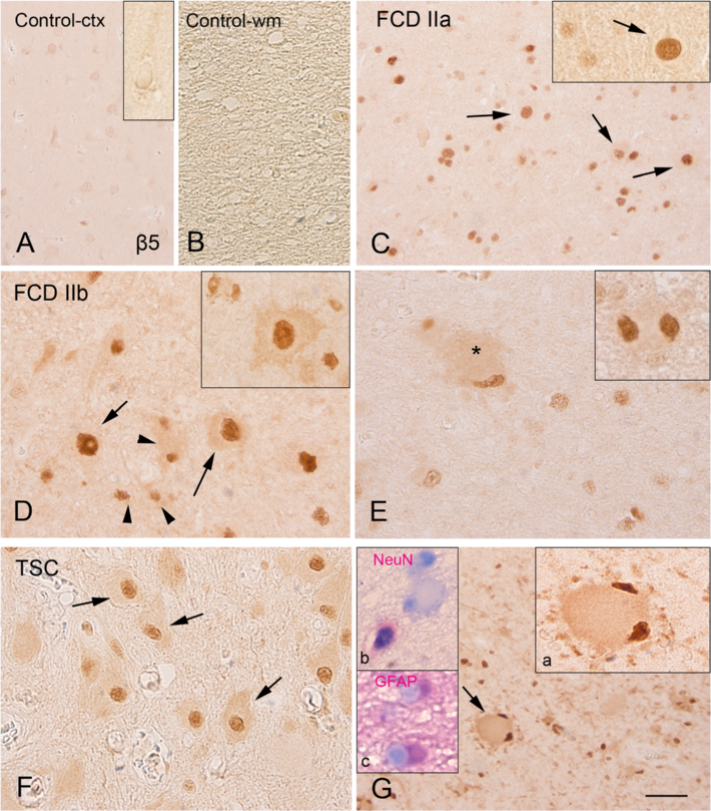
β5 proteasome subunit immunoreactivity in control, focal cortical dysplasia (FCD) type IIa, FCD type IIb, and tuberous sclerosis complex (TSC). Panels **a** and **b**: (control) show the weak immunoreactivity in both the cortex (**a** insert: neuron) and white matter (**b** not detectable glial expression). Panel **c** (FCD IIa) shows positive dysmorphic neurons (*arrows*; insert: high magnification, nuclear expression). Panels **d** and **e** (FCD IIb) show several β5 positive cells within the cortex **(d)** and white matter **(e)**, including dysmorphic neurons (*arrows* in **d** and insert, nuclear and cytoplasmic expression), glial cells (*arrow heads* and insert in **d**), and balloon cells (*asterisk* in **e**; insert: high magnification, with prominent nuclear expression). Panels **f** and **g** (TSC-tuber): β5 subunit expression is observed within the tuber in dysmorphic neurons (*arrows* in **f**; insert **b** in panel **g**: co-localization with the neuronal marker NeuN; insert **c** in panel **g**, co-localization with GFAP) and in giant cells (*arrow* in **g** and insert **a**; insert **b**: co-localization with NeuN; insert **c**: expression in glial cells, co-localization with GFAP). The inserts within panels show images of the different cell types at higher magnification in separate specimens. Scale bar in **g**: **a–c, g**: 80 μm; **d–f**: 40 μm

**Fig. 4:**
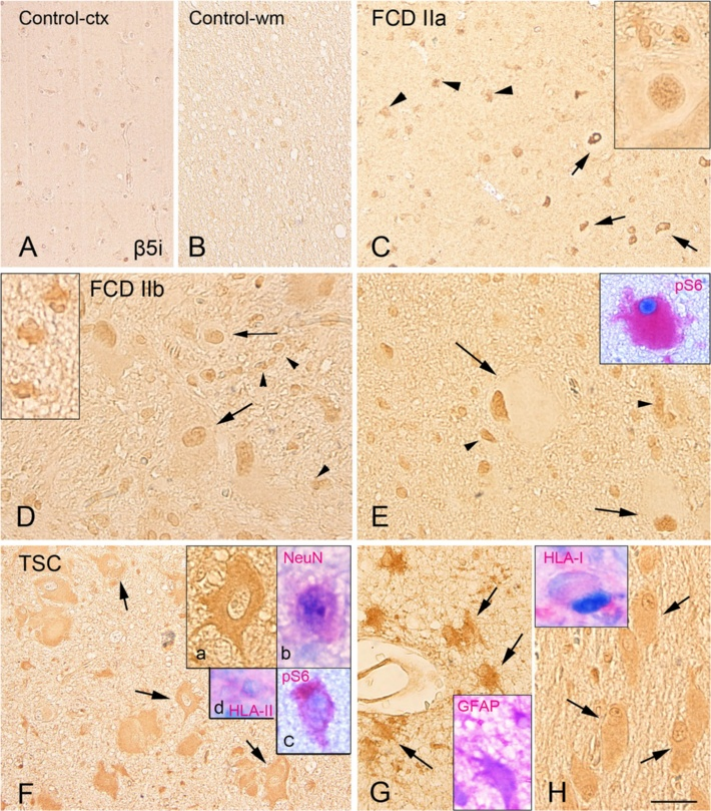
β5i proteasome subunit immunoreactivity in control, focal cortical dysplasia (FCD) type IIa, FCD type IIb, and tuberous sclerosis complex (TSC). Panels **a** and **b**: control cortex **(a)** and with white matter **(b)** with weak β5i expression. Panel **c** (FCD IIa) shows expression within the dysplastic region with positive dysmorphic neurons (*arrows* and insert, nuclear expression) and glial cells (*arrow heads*). Panels **d** and **e** (FCD IIb) show several β5i-positive cells within the cortex **(d)** and white matter **(e)**, including dysmorphic neurons (*arrow* in **d**), glial cells (*arrow heads* **d** and **e**; insert in **d**), and balloon cells (*arrows* in **e**; insert: co-localization with pS6). Panels **f**, and **g** (TSC-tuber): strong β5i subunit expression is observed within the tuber in dysmorphic neurons (**f** arrows and inserts **a** and **b**; **b** co-localization with the neuronal marker NeuN; **c** co-localization with the pS6; **d** co-localization with HLA-II), glial cells (*arrows* in **g**; insert in **g**, co-localization with GFAP), and in giant cells (*arrows* in **h**; insert: co-localization with HLA-I). The inserts within the panels show images of the different cell types at higher magnification in separate specimens. Scale bar in **h**: **a–c** 80 μm; **d–h** 40 μm

The clinical features of the cases included in this study are summarized in Table 1. All operated patients had a history of chronic pharmacoresistant epilepsy. In this study, we included patients with mild degree of cortical dysplasia (mMCDs; [33]). Age at surgery, seizure duration, and seizure frequency were not statistically different between patients with FCD II and mMCD in this cohort, as well as between the FCD IIa and FCD IIb cases included in our cohort. Accordingly to the international consensus classification system of FCD [21], FCD II represents isolated focal lesions with architectural and dysmorphic abnormalities (FCD IIa with dysmorphic neurons only; FCD IIb with dysmorphic neurons and balloon cells; Figs. 1, 2, 3, and 4c–e). TSC patients were younger compared to mMCD and FCD patients. All six TSC tubers displayed similar histopathological features, including loss of lamination, astrogliosis, dysmorphic neurons, and giant cells with pale eosinophilic cytoplasm ([34]; Figs. 1, 2, 3, and 4f, g).

### Proteasome subunit expression in FCD and cortical tubers

Expression of β1, β1i, β5, and β5i was observed in FCD, cortical tubers, and mMCD specimens (Figs. 1, 2, 3, and 4; Additional file 2: Figure S2 and Additional file 4: Figure S4). We observed differences in the expression level as well as in the cell-specific and subcellular distribution of the different subunits (Table 3).

#### Constitutive proteasome catalytic subunit β1 and β5

Moderate expression of β1 and β5 subunits was observed in human control cortical specimens (Figs. 1a, b and 3a, b; Table 3). Nuclear neuronal expression was detected for β5 in MCD specimens (Table 3; Additional file 4: Figure S4E); whereas only cytoplasm expression was detected in specimens from patients with Alzheimer’s disease for both subunits (Additional file 4: Figure S4B, F). Increased expression of both constitutive subunits was observed in FCD and TSC specimens (Figs. 1c–g and 3c–g; Table 2). In the large majority of FCD and TSC cases, β1 IR was detected in the cytoplasm and nucleus of neuronal and glial cells (Fig. 1c–g; Table 3). β1 was also detected in the balloon (FCD IIb; Fig. 1e) and giant cells (TSC; Fig. 1g). FCD and TSC specimens displayed also strong β5 IR with prominent nuclear expression in both neuronal and glial cells, as well as in the balloon (FCD IIb) and giant cells (TSC; Fig. 3c–g; Table 3). A similar pattern was detected in the postmortem TSC case; double-labeling experiments confirmed the co-localization with astroglial and neuronal markers within the dysplastic area for both subunits in FCD and TSC specimens (Figs. 1f–g and 3g).

#### Immunoproteasome subunits β1i and β5i

In the large majority of control (Figs. 2a, b and 4a, b) and mMCD (Additional file 4: Figure S4C, G) specimens, the immunoproteasome subunits β1i and β5i were under the detection levels in both neuronal and glial cells (Table 3). β1i and β5i were consistently high in FCD and TSC specimens (Table 3; Additional file 2: Figure S2) with strong cytoplasmic and nuclear IR in neuronal and glial cells, in both surgical postmortem TSC specimens (whereas only cytoplasmic expression was detected in glial cells in specimens from patients with Alzheimer’s disease for both β1i and β5i subunits; Additional file 4: Figure S4D, H). A similar pattern with strong expression in FCD and TSC specimens was observed using in situ hybridization (Additional file 5: Figure S5). Double-labeling experiments confirmed the co-localization with astroglial and neuronal markers, as well as with major histocompatibility complex (MHC) class I (HLA-I; in few balloon/giant cells and in dysmorphic neurons) within the dysplastic area for both subunits in FCD and TSC specimens (Figs. 2 and 4f, g). In regions with prominent activation of microglia, IR for both β1i and β5i was also observed in cells of the microglia/macrophage lineage (HLA-II; Figs. 2 and 4). The balloon (FCD IIb; Fig. 2e) and giant cells (TSC; Fig. 4g) displayed β1i and β5i IR as well (Table 2). Co-localization was observed for both β1i and β5i with pS6 (Figs. 2 and 4). β1i and β5i expression in neurons was positively associated with pS6 expression within our MCD cohort (β1i cytoplasm, *r* = 0.5905, *p* = 0.030; β1i nucleus, *r* = 0.6244, *p* = 0.0014; β5i cytoplasm, *r* = 0.4510, *p* = 0.0065). A positive correlation was detected between β1i and β5i expression in neurons and glial cells and IL-1β IRS within the dysplastic region (neuronal β1i cytoplasm, *r* = 0.4287, *p* = 0.0413; neuronal β1i nucleus, *r* = 0.5090, *p* = 0.0131; glia β1i cytoplasm, *r* = 0.5298, *p* = 0.0093; glia β1i nucleus, *r* = 0.6091, *p* = 0.0003; neuronal β5i cytoplasm, *r* = 0.7322, *p* = <0.001; glia β5i cytoplasm, *r* = 0.7005, *p* = <0.001; glia β5i nucleus, *r* = 0.4210, *p* = 0.0455).

#### Immunoproteasome subunit expression and clinical features

We found no statistically significant association between the IRS of β1, β1i, β5 or β5i, and clinical features, such gender, age at surgery, location of the lesion, or duration of epilepsy. However, a positive correlation was observed between nuclear glial and neuronal subunit expression and the pre-operative seizure frequency (β1 and β1i neuron *τ* = 0.639 and *τ* = 0.633, *p* < 0.001; β1 and β1i glia *τ* = 0.479, *p* = 0.005 and *τ* = 0.65, *p* < 0.001; β1 and β1i neuron *τ* = 0.550, *p* = 0.004 and *τ* = 0.417, *p* = 0.016; β1 and β1i glia *τ* = 0.570, *p* = 0.001; *τ* = 0.586, *p* = 0.001).

#### Regulation of immunoproteasome subunit expression in human glial cells in culture

**Fig. 5:**
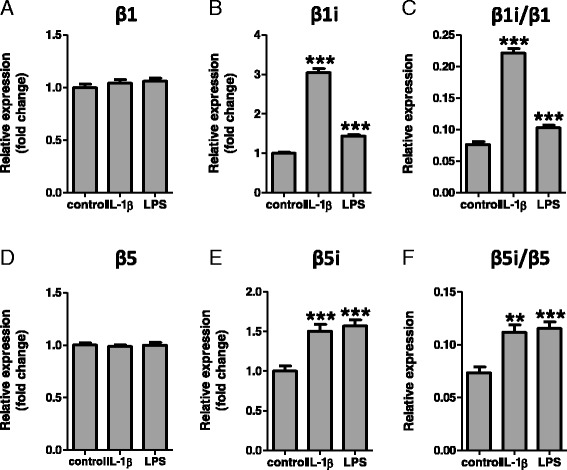
Effects of IL-1β and LPS stimulation on immunoproteasome subunit expression in cell culture. Quantitative real-time PCR of proteasome expression in human fetal astrocytes after the 24 h exposure to IL-1β (10 ng/ml) or LPS (100 ng/ml). **a**, **b**, **d**, **e** Stimulation with IL-1β or LPS increased expression of the β1i **(b)** and the β5i **(e)** subunits compared to control, but not of the constitutive β1 **(a)** and β5 **(d)** subunits. **c, f** Stimulation with either IL-1β or LPS increased the β1i/β1 **(c)** and the β5i/β5 **(f)** ratios. Data are expressed relative to the levels observed in untreated cells and are mean ± SEM (*n* = 5). **p < 0.01, ***p < 0.001 compared to control, Mann-Whitney *U* test

**Fig. 6:**
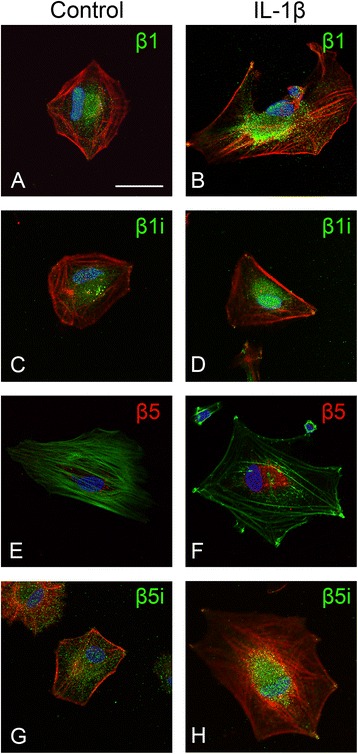
Effects of IL-1β stimulation on proteasome subunit expression in astrocytes in cell culture. Expression of β1 (**a** and **b**; *green*), β1i (**c** and **d**; *green*), β5 (**e** and **f**; *red*), and β5i (**g** and **h**; *green*) in unstimulated human fetal astrocytes (*left panels*) and in astrocytes after exposure to IL-1β (24 h; 10 ng/ml, *right panels*); increased expression of all subunits was observed. A translocation of particularly the β1i and β5i subunits, shifting from cytoplasmic to perinuclear-nuclear expression following IL-1β treatment was observed. Cells were counterstained with phalloidin (actin filaments; *red* in **a–d** and **g–h**, *green* in **e** and **f**) and diamidino-2-phenylindole, DAPI (nuclei; *blue*). Scale bar in **a**: 15 μm

Since IL-1β is known to be strongly upregulated in FCD and TSC human brain specimens [27, 34, 35] and to play a key pathogenic role in human epilepsy (for review, see [20, 36]; we also investigated whether this inflammatory cytokine could play a role in the regulation of the expression and cellular localization of immunoproteasome subunits. qPCR analysis of astrocyte-enriched human fetal cell cultures demonstrated that exposure to IL-1β did not modify the expression of the constitutive subunits (Fig. 5a, d) but did consistently increase the expression of both immunoproteasome subunits β1i and β5i (Fig. 5b, e), increasing the β1/ β1i and β5/β5i ratios (Fig. 5c, f). Treatment with LPS, also a potent inducer of the immune response, gave comparable results (Fig. 5). Exposure to IL-1β and LPS did not significantly affect the expression of IFNγ in these cultures. Immunohistochemistry showed a translocation of the β1i and β5i subunits, shifting from cytoplasmic to perinuclear-nuclear expression following IL-1β treatment (Fig. 6d, h).

#### Effects of rapamycin on proteasome subunit expression in FCD II-derived astrocytes

**Fig. 7:**
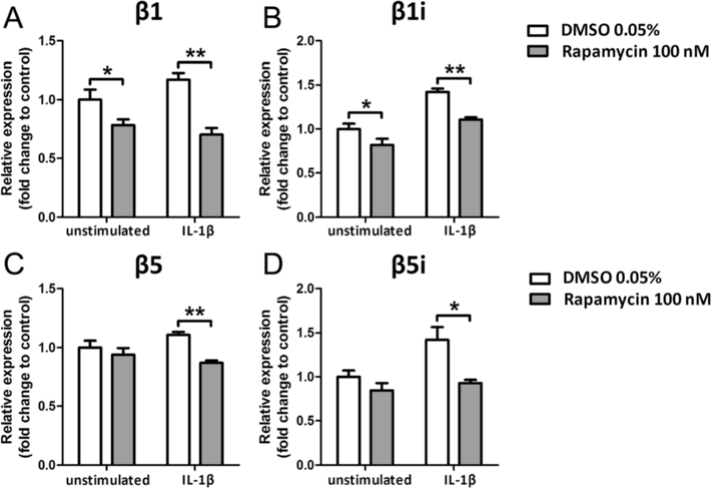
Effects of rapamycin on proteasome subunit expression in astrocytes derived from FCD type II. Quantitative real-time PCR of proteasome expression in human FCD cells after 48 h treatment with 100 nM rapamycin, under basal and stimulated (IL-1β 10 ng/ml) conditions. **a**, **b** Treatment with rapamycin decreased the expression of β1 **(a)** and β1i **(b)** subunit, both in the basal and under stimulated conditions. **c, d** Treatment with rapamycin decreased the expression of β5 **(c)** and β5i **(d)** subunit under stimulated, but not in basal conditions. Data are expressed relative to the levels observed in untreated cells and are mean ± SEM (*n* = 5). *p < 0.05, **p < 0.01 compared to control, Mann-Whitney *U* test

**Fig. 8:**
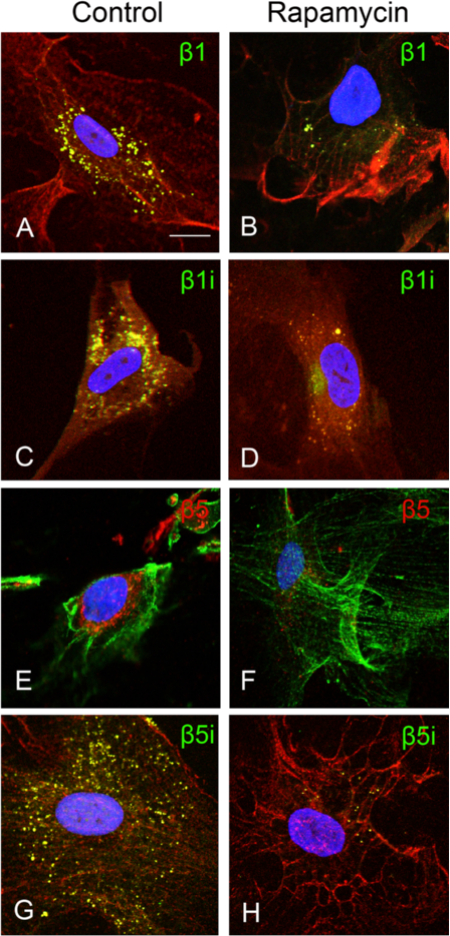
Effects of rapamycin treatment on proteasome subunit expression in FCD type II derived cells. Expression of β1 (**a** and **b**; *green*), β1i (**c** and **d**; *green*), β5 (**e** and **f**; *red*), and β5i (**g** and **h**; *green*) in untreated FCD cells (*left panels*) and in FCD cells after 48 h of treatment with 100 nM rapamycin (*right panels*). Expression of all subunits was decreased after treatment with rapamycin. Cells were counterstained with phalloidin (actin filaments; *red* in **a–d** and **g–h**, *green* in **e** and **f**) and diamidino-2-phenylindole, DAPI (nuclei; *blue*). Scale bar in **a**: 15 μm

Since both FCD II and TSC are associated with constitutive activation of the mTOR pathway [19, 20], we investigated whether the canonical and allosteric mTOR kinase rapamycin modulates the immunoproteasome in cell cultures derived from FCD II specimens. Western blot analysis confirmed that 100 nM rapamycin reduced the phosphorylation of S6 (pS6) in human astrocytes, an indicator of mTOR activation (Additional file 3: Figure S3). Pretreatment with 100 nM rapamycin reduced the mRNA expression of β1 and β1i subunits under both unstimulated and stimulated conditions (Fig. 7a, b). β5 and β5i expression was reduced by rapamycin in the presence of IL-1β (Fig. 7c, d). Immunocytochemical analysis of these FCD cells confirmed the negative modulation of proteasome subunits by rapamycin (Fig. 8).

## Discussion

The present study reports in detail the expression pattern and cellular localization of the constitutive and immunoproteasome subunits in FCD II and TSC cortical tubers and mMCD. The cell-specific distribution of proteasome subunits in relation with the epileptogenicity of these developmental lesions as well as their regulation in human astrocytes is discussed in the following paragraphs.

### Proteasome subunits expression in malformations of cortical development: prominent expression in FCD II and TSC

Our data show prominent expression of both constitutive and immunoproteasome subunits in MCD, such as FCD and TSC, associated with the mTOR pathway. In all the FCD II and TSC specimens examined, the IR for β1, β1, β5, and β5i was increased within the dysplastic regions where prominent gliosis and the presence of dysmorphic neurons and balloon or giant cells (in FCD IIb and TSC, respectively) was observed. Constitutive and particularly immunoproteasome subunits displayed increased expression compared to control but also compared to mMCD specimens from patients with chronic epilepsy. These results indicate that increased expression of proteasome subunits is not simply an effect of seizure activity; moreover, the duration of epilepsy in mMCD cases did not differ from FCDs and was even longer compared to TSC cases. However, a positive correlation was observed between nuclear glial and neuronal proteasome subunit expression and the pre-operative seizure frequency. We acknowledge limitations to the interpretation of these results; therefore, an evaluation of the real biological contribution of proteasome subunit expression to seizure generation and frequency deserves further investigation in experimental models.

Several proteasome subunits show nuclear localization signaling [37], and previous studies in the human brain indicate that proteasomes are expressed in both cytoplasm and nuclei of different cell types, including glial and neuronal cells [24, 38]. Immunoproteasome expression restricted to nuclei of astrocytes has been reported in the brain after an infection with lymphocytic choriomeningitis virus, suggesting involvement of the nuclear envelope in the compartmentalization of immature proteasome precursors [39]. Whether the nuclear proteasome subunits represent (as suggested by Kremer et al. [39]) immature proteasome precursors or are proteolytically active remains still to be investigated. The nuclear proteasome subunit accumulation may reflect the induction of the proteasome system under conditions associated with cell injury and inflammation with the possibility of nucleo-cytoplasmic transfer in cells, as glial cells, undergoing cell division or during apoptosis [37]. However, the β1i subunit in the nuclear-enriched fraction has also been detected in its catalytically active form [40], and several studies indicate a possible functional role of the immunoproteasome in transcriptional regulation [41–43]. The expression pattern, either nuclear or cytoplasmic proteasome expression, can be influenced by the type and duration of fixation [37]. However, similar pattern was observed in surgical and postmortem TSC brain tissue.

One of the major regulatory factors of immunoproteasome induction is inflammation [43, 44]. Several studies confirmed the occurrence of complex inflammatory changes, involving both glial and neuronal cells, and the activation of the IL-1β pathway, particularly in FCD II and TSC [20, 34, 35, 45–48]. Thus, the pro-inflammatory environment may contribute to the activation of the proteasome system, particularly to the induction and expression of the immunoproteasome subunits. Accordingly, our in vitro studies in human astrocytes and FCD cultures indicate that IL-1β treatment increases the induction of, in particular, the immunoproteasome subunits β1i and β5i, with the increase of their perinuclear-nuclear localization. This observation supports the role of astrocytes as targets of regulation of the immunoproteasome under various conditions associated with the activation of the IL-1β pathway [16] and indicates that pro-inflammatory cytokines, other than IFNγ, may regulate immunoproteasome expression. Activation of inflammatory pathways, including IL-1β, may also play a role in the regulation of immunoproteasome expression in other cell types, such as neurons. Accordingly, we found a positive correlation between the expression of immunoproteasome subunits in both glial and neuronal cells and the expression of IL-1β within the dysplastic area in FCD II and in TSC specimens. Moreover, increasing evidence supports the role of the immunoproteasome in the activation of the NF-kB pathway, modulation of pro-inflammatory cytokine production, and oxidative stress response [9, 43, 49–52]. Induction of the β5i subunit has also been shown in vivo following activation of the Toll-like receptor 4 (TLR4)-mediated NF-kB signaling pathway by LPS [53]. Thus, we may speculate about the existence of a reinforcing feedback loop between NF-kB pathway and the immunoproteasome system, which may play a crucial role in perpetuating the pro-epileptogenic inflammatory response in epilepsy. Interestingly, Mishto et al. [18] provide additional experimental evidence of the regulation of β5i subunit by TLR4 signaling in epileptogenic tissue.

The immunoproteasome is known to improve MHC class I (MHC-I) antigen presentation and has been suggested to have a central function at the interface between the innate and adaptive immune system (reviewed in [11]). Interestingly, FCD II and TSC specimens are characterized by prominent activation of both innate and adaptive immune responses (for review, see [20, 36]). Moreover, recent studies provide evidence of an upregulation of MHC-I, involving also balloons/giant cells and neurons, in both FCD II and TSC specimens [54].

FCD II and TSC cases are characterized by architectural or cellular changes associated with mTOR pathway activation [20, 21]. The innate and adaptive immune responses have also been shown to be influenced by the mTOR pathway [55–57]. Moreover, the mTOR complex 1 (mTORC1) has been identified as a key regulator of autophagy [58, 59], a pathway which is defective in FCD II and TSC [60]. Increasing evidence indicates a strong relationship with tight coordination between the autophagy and the proteasome system [61]. Thus, we cannot exclude a role of mTOR in the regulation of the proteasome system, including immunoproteasome subunit expression. Accordingly, we observed a positive correlation between immunoproteasome subunit expression in neurons and pS6 expression, indicating the activation of the mTOR signal transduction pathway. The relationship between mTOR and proteasome system is also supported by the in vitro experiments showing that inhibition of the mTOR pathway by the potent allosteric mTORC1 inhibitor rapamycin was able to reduce the level of expression of inducible proteasome subunits in FCD-derived cells. This is in agreement with a recent study showing reduction of the immunoproteasome by rapamycin in H9c2 cells as well as in mouse heart in vivo [62]. Evaluation of the possible effect of rapamycin on the expression of the brain immunoproteasome in vivo deserves further studies and is presently under investigation [63].

### Immunoproteasome inhibition as therapeutic strategy?

An example of the possible use of inhibition of the immunoproteasome as therapeutic strategy in epilepsy is represented by the study of Mishto and colleagues [18] in which specific inhibition of the β5i subunit by ONX-0914 [64] resulted in prevention, or significant delay, of 4-aminopyridine-induced seizure-like events in acute rat hippocampal/entorhinal cortex slices, particularly in slices of epileptic rats. Clinically approved proteasome inhibitors targeting the catalytic activity of both the constitutive proteasome and the immunoproteasome have been already used in hematological malignancies [65–67]. New-generation small molecules specifically targeting the immunoproteasome are under clinical development and have been already evaluated in a large variety of animal models of autoimmune diseases and proposed as novel therapeutic approaches for patient with multiple sclerosis, as well as in neurodegenerative diseases (for reviews, see [16, 68, 69]).

However, recently alternative functions for the immunoproteasome have also been considered, suggesting that the induction of the immunoproteasome may also play a role in neuronal protection and repair after injury, contributing to the preservation of cell viability upon cytokine-induced oxidative stress [49, 70, 71], which is known to be increased within the TSC tubers [72]. In particular, evidence has been provided that the immunoproteasome plays a role in the clearance of damaged proteins accumulating upon inflammation or oxidative stress (for review, see [49]), which are also detected in TSC and FCD [73]. Accordingly, the formation of aggresome-like-induced structures and increased sensitivity to apoptosis has been reported in immunoproteasome deficiency in cells and in a murine inflammation model [49, 71]. Additional studies support alternative physiological function of the immunoproteasome subunits, including also cell proliferation, cell signaling, and synaptic remodeling (for review, see [49, 74, 75]). Thus, an effective therapeutic intervention based on the immunoproteasome has to take into consideration the preservation of the potential beneficial functions of its activation, particularly during brain development.

## Conclusions

One important question is whether the activation of the immunoproteasome system in the brain tissue may per se be responsible for an increased susceptibility to seizure activity observed in FCD and TSC. As discussed above, experimental studies in the hippocampal/entorhinal cortex slices suggest that the pharmacological inhibition of the β5i subunit may modulate seizure activity. Whether these findings can be translated to other experimental models, including models of FCD and TSC, deserves further investigation.

To conclude, our observations support the occurrence of a prominent deregulation of the proteasome system in MCD. In particular, the induction of immunoproteasome subunits in both glial and neuronal cells appears to be a feature of FCD II and TSC and may represent an important accompanying feature of the immune response in these developmental lesions. Therefore, understanding the role of the immunoproteasome in epilepsy-associated pathologies may have great importance in view of the development of new therapeutic strategies.

## Additional files

Additional file 1: Figure S1. Representative immunoblot analysis of total homogenates from (*n* = 3) surgical hippocampal specimens; β-subunits (β1, ~25 kDa; β1i, ~22 kDa; β5, ~25 kDa; β5i, ~25 kDa; β-actin ~42 kDa). (JPG 134 kb) Additional file 2: Figure S2. β1i and β5i intensity signal in control, mMCD, FCDII, and TSC. FCD: focal cortical dysplasia; TSC: tuberous sclerosis complex; mMCD: mild malformations of cortical development. (PPTX 61 kb) Additional file 3: Figure S3. Effect of the different treatments on fetal astrocyte cell cultures. A: scatterplots of eFluor viability dye staining as analyzed by flow cytometry after different treatments. B: Quantification of viable cells based on eFluor viability staining. Neither treatment with IL-1β nor rapamycin negatively influenced viability of cell cultures. C: Western blot analysis showed effective reduction of phosphorylated S6 after 24 h of 100 nM rapamycin treatment. FSC: forward scatter. (JPG 1420 kb) Additional file 4: Figure S4. Proteasome subunit immunoreactivity (β1, β1i β5, and β5i) in mild MCD (mMCD) and in Alzheimer’ s disease (Alz). Panels A, C, E, and G: mMCD. A: low β1 expression (insert: high magnification of a neuron, with weak nuclear expression). C: low β1i expression (insert: high magnification of a neuron). E: nuclear expression of β5 (arrows; neuron in insert). G: low β5i expression (neuron in insert). Panels B, D, F, and H (Alz; hippocampus). B: β1 expression in neurons (CA1; arrows, cytoplasmic expression) and around amyloid plaques (arrow-heads); D: β1i expression in glial cells (arrows, cytoplasmic expression). F: low β5 expression in neuronal cells (arrows). H: β5i expression in glial cells (arrows, cytoplasmic expression). Scale bar in B: A, C, F, and G: 80 μm; B, D, and H: 40 μm. (JPG 2799 kb) Additional file 5: Figure S5. In situ hybridization of β1i and β5i, proteasome subunit immunoreactivity in control, focal cortical dysplasia (FCD) type IIb, and tuberous sclerosis complex (TSC). Panels A–D: control cortex (A–C) and with matter (B–D); β1i (A–B) and β5i (C–D). Panels E–F (FCD IIb) and panels C–G (TSC) shows strong expression within the dysplastic region with several positive dysmorphic neurons (arrows and inserts in E (a) and F), giant cells (inserts in G and H (a)), and glial cells (inserts (b) in E and H). Scale bar in H: A–H: 80 μm. (JPG 3206 kb) Additional file 6: Supplementary methods: flow cytometr﻿ic analysis,* in situ* hybridization, Western blot analysis and image quantification. (DOC 46 kb)

## Abbreviations

FCD: Focal cortical dysplasia
IRS: Immunoreactivity score
MCD: Malformations of cortical development
MTLE: Mesial temporal lobe epilepsy
PBS: Phosphate-buffered saline
TSC: Tuberous sclerosis complex
