# Supplementary figures and images for: Dysregulation of the (immuno)proteasome pathway in malformations of cortical development
Source: J Neuroinflammation. 2016 Aug 26;13(1):202. doi: 10.1186/s12974-016-0662-z (PMC5002182; doi:10.1186/s12974-016-0662-z)

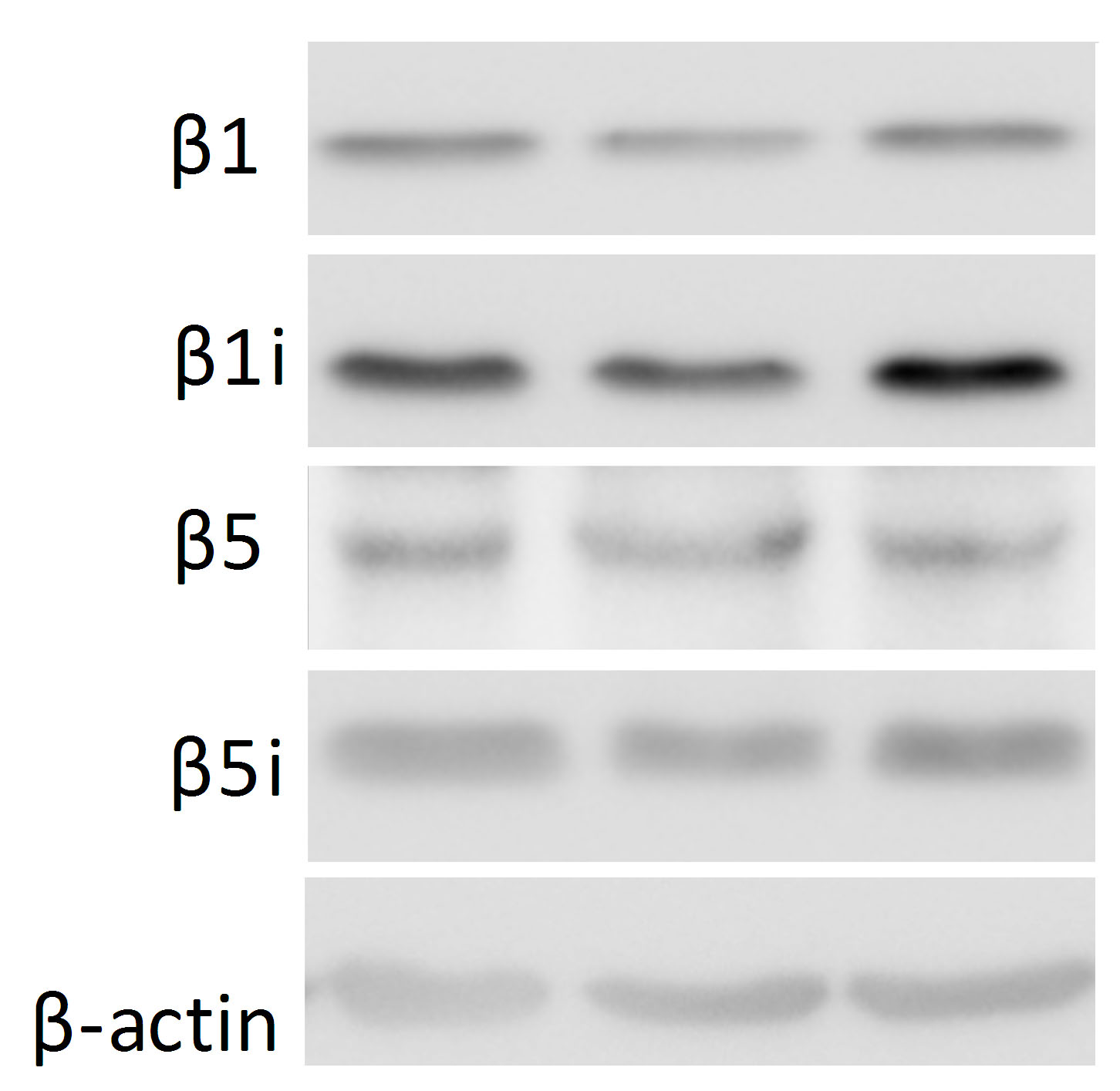

Supplement: Additional file 1: Figure S1. — Representative immunoblot analysis of total homogenates from (n = 3) surgical hippocampal specimens; β-subunits (β1, ~25 kDa; β1i, ~22 kDa; β5, ~25 kDa; β5i, ~25 kDa; β-actin ~42 kDa). (JPG 134 kb) [file 12974_2016_662_MOESM1_ESM.jpg]

## Slide 1
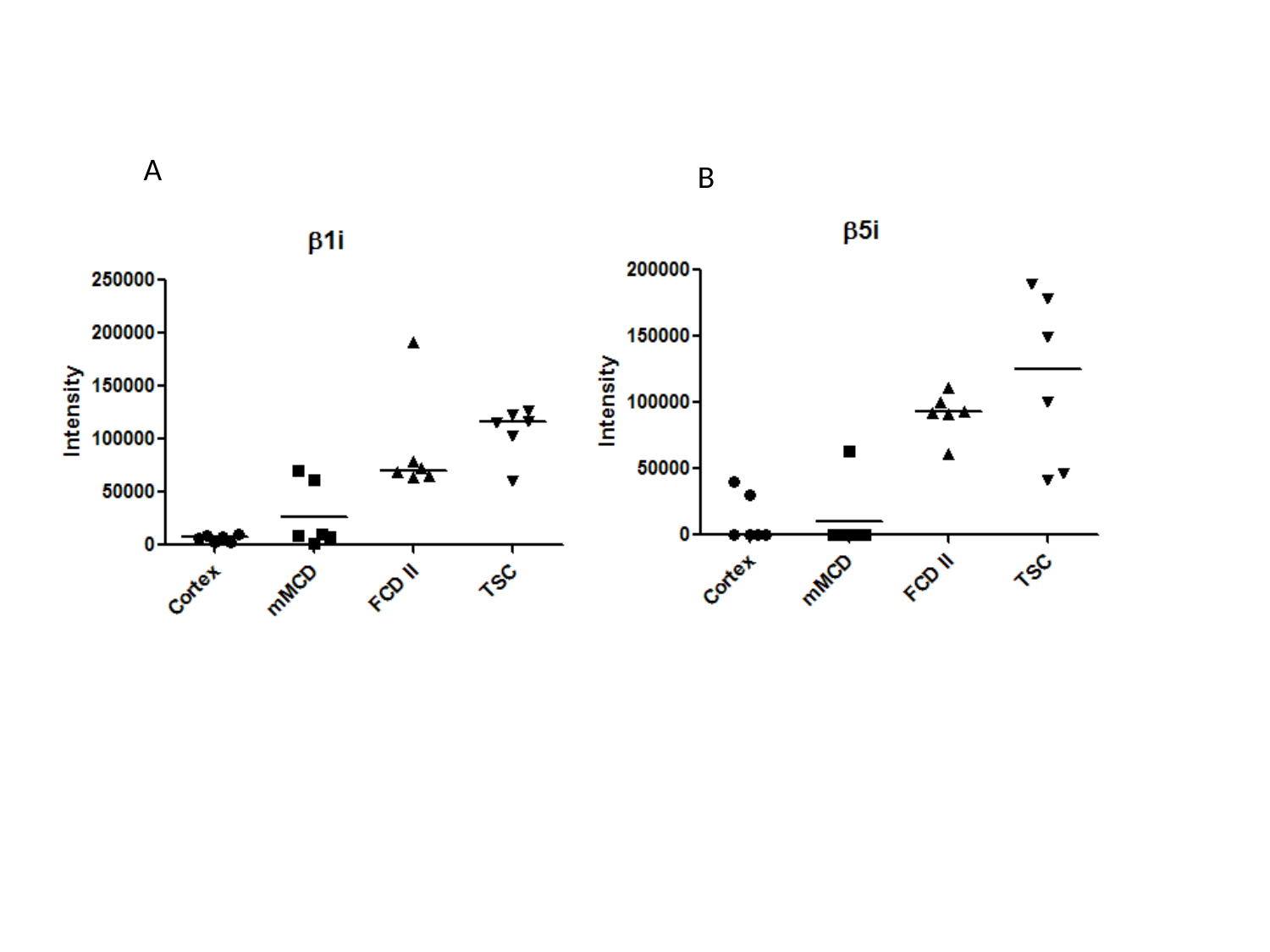

A
B

Supplement: Additional file 2: Figure S2. — β1i and β5i intensity signal in control, mMCD, FCDII, and TSC. FCD: focal cortical dysplasia; TSC: tuberous sclerosis complex; mMCD: mild malformations of cortical development. (PPTX 61 kb) [file 12974_2016_662_MOESM2_ESM.pptx]

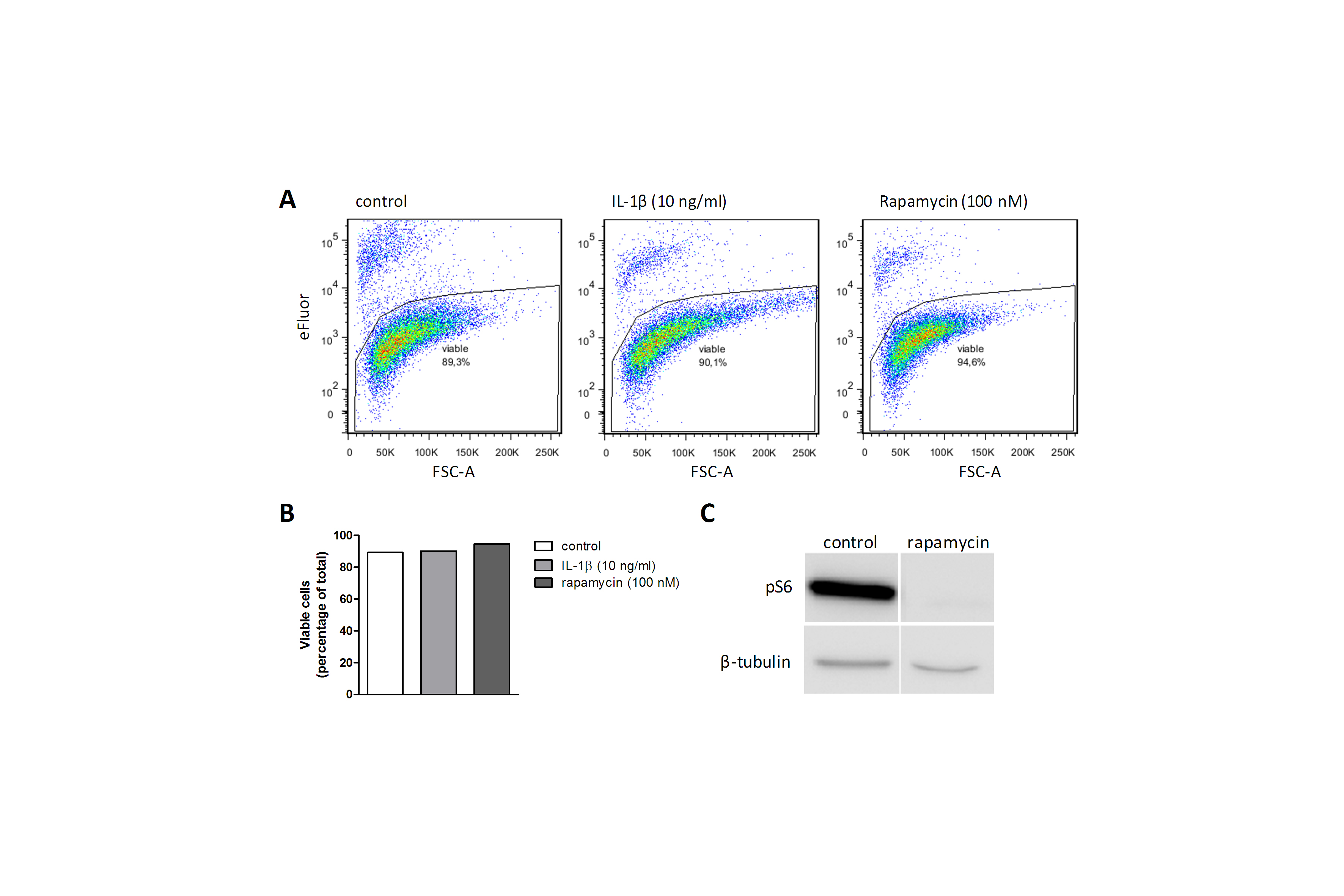

Supplement: Additional file 3: Figure S3. — Effect of the different treatments on fetal astrocyte cell cultures. A: scatterplots of eFluor viability dye staining as analyzed by flow cytometry after different treatments. B: Quantification of viable cells based on eFluor viability staining. Neither treatment with IL-1β nor rapamycin negatively influenced viability of cell cultures. C: Western blot analysis showed effective reduction of phosphorylated S6 after 24 h of 100 nM rapamycin treatment. FSC: forward scatter. (JPG 1420 kb) [file 12974_2016_662_MOESM3_ESM.jpg]

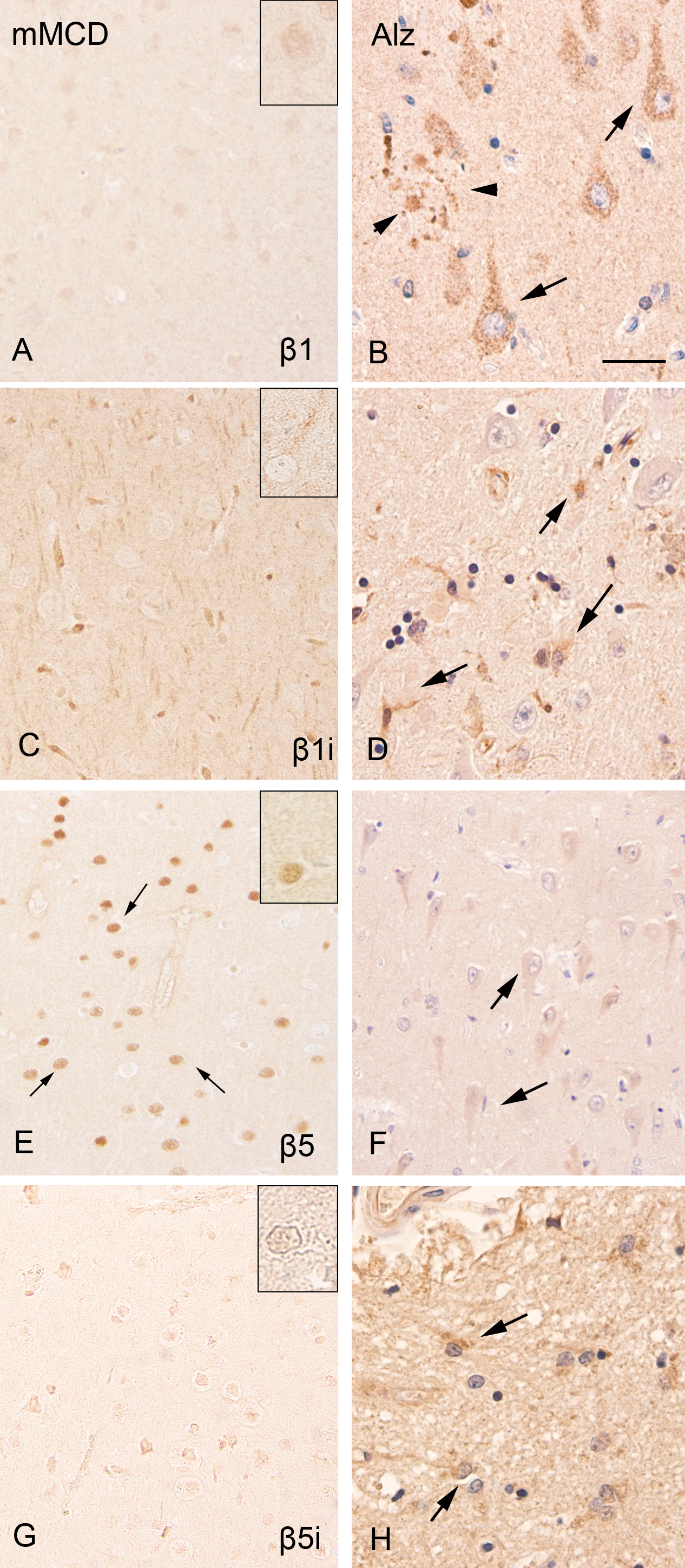

Supplement: Additional file 4: Figure S4. — Proteasome subunit immunoreactivity (β1, β1i β5, and β5i) in mild MCD (mMCD) and in Alzheimer’ s disease (Alz). Panels A, C, E, and G: mMCD. A: low β1 expression (insert: high magnification of a neuron, with weak nuclear expression). C: low β1i expression (insert: high magnification of a neuron). E: nuclear expression of β5 (arrows; neuron in insert). G: low β5i expression (neuron in insert). Panels B, D, F, and H (Alz; hippocampus). B: β1 expression in neurons (CA1; arrows, cytoplasmic expression) and around amyloid plaques (arrow-heads); D: β1i expression in glial cells (arrows, cytoplasmic expression). F: low β5 expression in neuronal cells (arrows). H: β5i expression in glial cells (arrows, cytoplasmic expression). Scale bar in B: A, C, F, and G: 80 μm; B, D, and H: 40 μm. (JPG 2799 kb) [file 12974_2016_662_MOESM4_ESM.jpg]

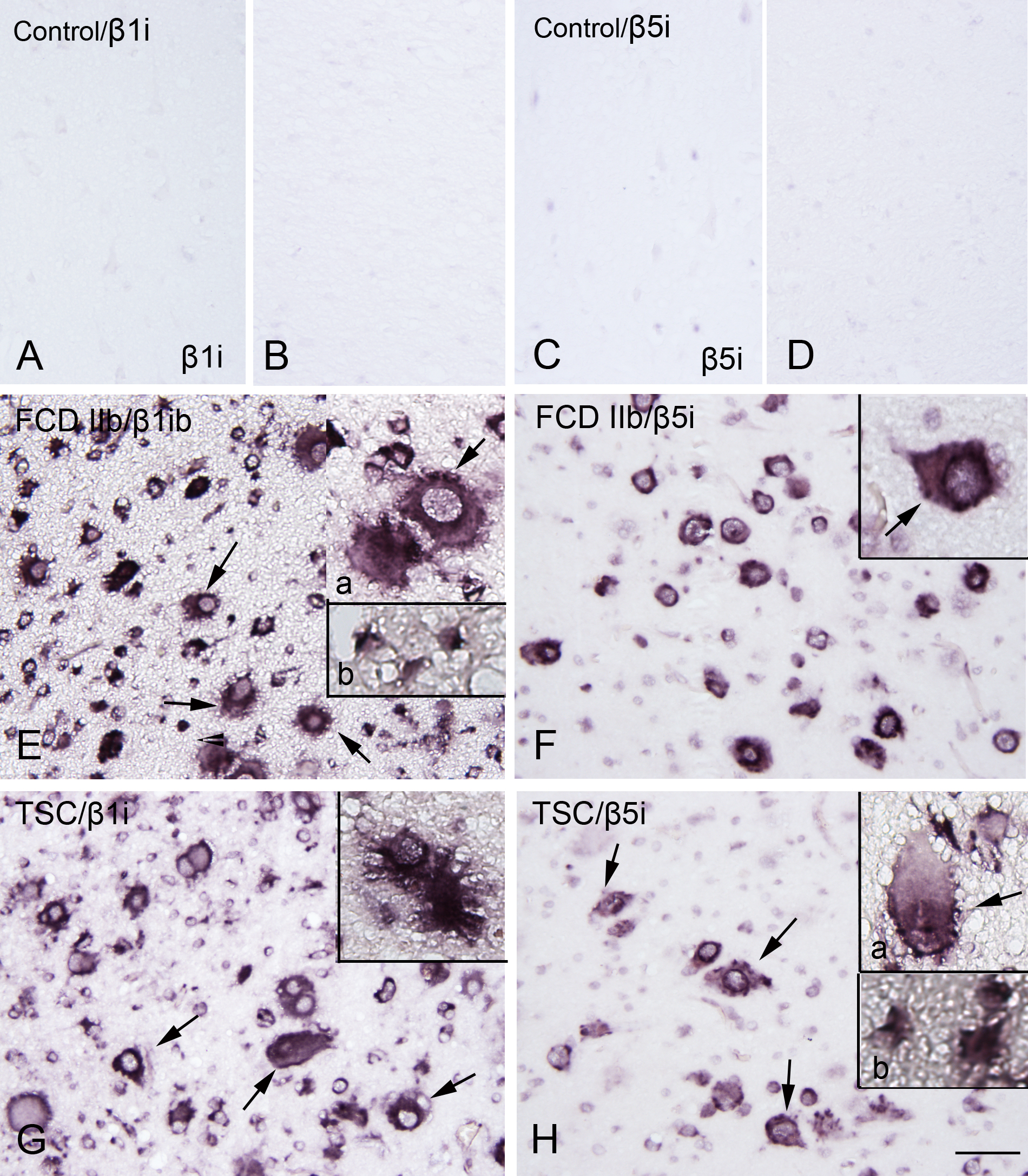

Supplement: Additional file 5: Figure S5. — In situ hybridization of β1i and β5i, proteasome subunit immunoreactivity in control, focal cortical dysplasia (FCD) type IIb, and tuberous sclerosis complex (TSC). Panels A–D: control cortex (A–C) and with matter (B–D); β1i (A–B) and β5i (C–D). Panels E–F (FCD IIb) and panels C–G (TSC) shows strong expression within the dysplastic region with several positive dysmorphic neurons (arrows and inserts in E (a) and F), giant cells (inserts in G and H (a)), and glial cells (inserts (b) in E and H). Scale bar in H: A–H: 80 μm. (JPG 3206 kb) [file 12974_2016_662_MOESM5_ESM.jpg]
