# Supplementary material for: Dysregulation of the (immuno)proteasome pathway in malformations of cortical development
Source: J Neuroinflammation. 2016 Aug 26;13(1):202. doi: 10.1186/s12974-016-0662-z (PMC5002182; doi:10.1186/s12974-016-0662-z)
Supplement: Additional file 6: — Supplementary methods: flow cytometr﻿ic analysis, in situ hybridization, Western blot analysis and image quantification. (DOC 46 kb) [file 12974_2016_662_MOESM6_ESM.doc]

**Supplementary**

**Flow cytometric analysis**

Viability of cell cultures was determined by flow cytometric analysis using Fixable Viability Dye eFluor® 780 (eBioscience, San Diego, CA, USA). Flow cytometric analysis of stained cells was performed using a FACSCanto Flow Cytometer equipped with FACSDiva software (BD Biosciences) and data analysis was performed using FlowJo 7.6 (FlowJo LLC, Ashland, OR, USA).

**Western blot analysis**

Frozen surgical hippocampal specimens or cells in culture were homogenized in lysis buffer containing 10 mM Tris (pH 8.0), 150 mM NaCl, 10% glycerol, 1% NP-40, 0.4 mg/ml Na-orthovanadate, 5 mM EDTA (pH 8.0), 5 mM NaF and protease inhibitors (cocktail tablets, Roche Diagnostics, Mannheim, Germany). Protein content was determined using the bicinchoninic acid method. For electrophoresis, equal amount of proteins (50 μg/lane) were separated by sodium dodecylsulfate-polyacrylamide gel electrophoresis (SDS-PAGE, 12% acrylamide). Separated proteins were transferred to nitrocellulose paper by electroblotting for 1 h and 30 min (BioRad, Transblot SD, Hercules, CA). After blocking for 1 h in TBST (20 mM Tris, 150 mM NaCl, 0.1% Tween, pH 7.5)/5% non-fat dry milk, blots were incubated overnight at 4°C with the primary antibodies (Table 2; proteasome β1, 1:1000; proteasome β1i, 1:1000; proteasome β5, 1:2500; proteasome β5i, 1:1000; β-actin, mouse monoclonal, Sigma, St. Louis, MO; 1: 10,000; β-tubulin, mouse monoclonal, Sigma, St. Louis, MO; 1: 2000). After several washes in TBST, the membranes were incubated in TBST / 5% non-fat dry milk, containing the goat anti-rabbit or rabbit anti-mouse antibodies coupled to horse radish peroxidase (1:2500; Dako, Denmark) for 1 h. After washes in TBST, immunoreactivity was visualized using ECL PLUS western blotting detection reagent (GE Healthcare Europe, Diegen, Belgium).

**Quantification**

The images were captured with an Olympus microscope (BX41, Tokyo, Japan) equipped with a digital camera (DFC500, Leica Microsystems-Switzerland Ltd., Heerbrugg, Switzerland). A total set of 6 images from 6 different cases were collected per pathology. Fiji (ImageJ2) was used for image processing. In a first step colour deconvolution (RGB colour space) was performed in order to separate positive cells from background according to the following channel parameters: red: 0.21408768, green: 0.8171735, blue: 0.4782719. Then a threshold (= 233) was applied and subsequently the images were converted to 8 bit gray-scale. The positive pixels/total assessed pixels, indicated as staining percentage area and intensity was calculated.

**In situ hybridization**

In situ hybridization (ISH) for β1i and β5i was performed using double digoxygenin (DIG) -labeled custom LNA oligonucleotides (Exiqon A/S, Denmark; see supplementary Table 1). The hybridizations were done on 5 µm sections of paraffin embedded materials as previously described [1]. The probes were hybridized at 56°C for 1 hour and the hybridization was detected with alkaline phosphatase (AP) labeled anti-DIG (Roche Applied Science, Basel, Switzerland). NBT (nitro-blue tetrazolium chloride)/BCIP (5-bromo-4-chloro-3'-indolyphosphate p-toluidine salt) was used as chromogenic substrate for AP. Negative control assays were performed without probes (sections were blank).

[1] Prabowo AS, van Scheppingen J, Iyer AM, Anink JJ, Spliet WG, van Rijen PC, et al. Differential expression and clinical significance of three inflammation-related microRNAs in gangliogliomas. J Neuroinflammation 12: 97.(2015).

**Legends supplementary figures**

**Figure1S. Representative immunoblot analysis of total homogenates from (n=3) surgical hippocampal specimens; β-subunits (β1, ~ 25 kDa; β1i,~ 22 kDa; β5, ~ 25 kDa; β5i, ~ 25 kDa; β-actin ~ 42 kDa).**

**Figure 2S. β1i and β5i intensity signal in control, mMCD, FCDII and TSC.**

**FCD: Focal Cortical Dysplasia; TSC: Tuberous Sclerosis Complex; mMCD: mild malformations of cortical development.**

**Figure 3S. Effect of the different treatments on fetal astrocyte cell cultures. A: scatterplots of eFluor viability dye staining as analyzed by flow cytometry after different treatments. B: quantification of viable cells based on eFluor viability staining. Neither treatment with IL-1β nor rapamycin negatively influenced viability of cell cultures. C: Western blot analysis showed effective reduction of phosphorylated S6 after 24 hours of 100 nM rapamycin treatment. FSC: forward scatter.**

**Figure 4S. Proteasome subunit immunoreactivity (β1, β1i β5 and β5i) in mild MCD (mMCD) and in Alzheimer’ s disease (Alz)**

Panels A,C,E,G: **mMCD**. **A:** low β1expression (insert: high magnification of a neuron, with light nuclear expression). **C:** low β1i expression (insert: high magnification of a neuron). **E:** nulclear expression of β5 (arrows; neuron in insert). **G:** low β5i expression (neuron in insert).

Panels B, D, F, H (**Alz;** hippocampus). **B:** β1 expression in neurons (CA1; arrows, cytoplasmic expression) and around amyloid plaques (arrow-heads); **D:** β1i expression in glial cells (arrows, cytoplasmic expression). **F:** low β5 expression in neuronal cells (arrows). **H:** β5i expression in glial cells (arrows, cytoplasmic expression). Scale bar in B: A,C, F,G: 80 µm; B,D,H: 40 µm.

**Figure 5S. *In situ* hybridization of β1i and β5i, proteasome subunit immunoreactivity in control, focal cortical dysplasia (FCD) type IIb and Tuberous Sclerosis Complex (TSC).**

**Panels A-D: control cortex (A-C) and with matter (B-D); β1i (A-B) and β5i (C-D) Panels E-F (FCD IIb) and panels C-G (TSC) shows strong expression within the dysplastic region with several positive dysmorphic neurons [arrows and inserts in E(a) and F], giant cells [inserts in G and H(a)] and glial cells [inserts (b) in E and H) Scale bar in H: A-H: 80 µm.**

Supplementary table 1

| **mRNA** | **Sequence** | **Hybridization temperature** |
| --- | --- | --- |
| β1i (PSMB9) | 5’DIG-+TmUmC+CmUmC+CmAmG+TmUmC+TmAmU+CmCmC+A -3’DIG | 56°C |
| β5i (PSMB8) | 5’DIG-+AmAmU+CmUmC+AmAmU+CmAmC+CmUmU+GmUmU+C -3’DIG | 56°C |

+ = LNA modification; m = 2’-O-methyl RNA base; DIG = digoxygenin label
